# Fast Virtual Fractional Flow Reserve Based Upon Steady-State Computational Fluid Dynamics Analysis: Results From the VIRTU-Fast Study

**DOI:** 10.1016/j.jacbts.2017.04.003

**Published:** 2017-08-28

**Authors:** Paul D. Morris, Daniel Alejandro Silva Soto, Jeroen F.A. Feher, Dan Rafiroiu, Angela Lungu, Susheel Varma, Patricia V. Lawford, D. Rodney Hose, Julian P. Gunn

**Affiliations:** a Department of Infection, Immunity and Cardiovascular Disease, University of Sheffield, Sheffield, United Kingdom; b Department of Cardiology, Sheffield Teaching Hospitals National Health Service Foundation Trust, Sheffield, United Kingdom; c Insigneo Institute for In Silico Medicine, University of Sheffield, Sheffield, United Kingdom; d Department of Electrotechnics and Electrical Measurements, Technical University of Cluj-Napoca, Cluj-Napoca, Romania

**Keywords:** computational fluid dynamics, coronary artery disease, coronary microvascular physiology, coronary modelling, coronary physiology, fractional flow reserve, virtual fractional flow reserve, CAD, coronary artery disease, CAG, coronary angiography, CFD, computational fluid dynamics, CMV, coronary microvasculature, FFR, fractional flow reserve, mFFR, invasively measured fractional flow reserve, PCI, percutaneous coronary intervention, RoCA, rotational coronary angiography, vFFR, virtual fractional flow reserve, vFFR_steady_, virtual fractional flow reserve computed with steady-state CFD analysis and cycle mean values, vFFR_trns_, virtual fractional flow reserve computed with full transient CFD, vFFR_ps-trns_, virtual fractional flow reserve computed with the pseudotransient steady-state method

## Abstract

Fractional flow reserve (FFR)-guided percutaneous intervention is superior to standard assessment but remains underused. The authors have developed a novel “pseudotransient” analysis protocol for computing virtual fractional flow reserve (vFFR) based upon angiographic images and steady-state computational fluid dynamics. This protocol generates vFFR results in 189 s (cf >24 h for transient analysis) using a desktop PC, with <1% error relative to that of full-transient computational fluid dynamics analysis. Sensitivity analysis demonstrated that physiological lesion significance was influenced less by coronary or lesion anatomy (33%) and more by microvascular physiology (59%). If coronary microvascular resistance can be estimated, vFFR can be accurately computed in less time than it takes to make invasive measurements.

## Highlights

• Computed vFFR promises the benefits of physiological lesion assessment without the drawbacks limiting use of the invasive method.
• Sophisticated, zero-dimension–coupled, transient, 3-dimensional CFD models provide high degrees of accuracy but are typically slow to compute, and models are sensitive to unknown physiological parameters such as myocardial resistance.
• Based on paired steady-state CFD analyses, 2 mathematical methods (“steady” and “pseudotransient”) were developed that accelerate the computation of vFFR from >36 h to <4 min.
• The pseudotransient method computed transient results, without the need for complex, and computationally expensive, full transient CFD analysis.
• Sensitivity analysis demonstrated that the hyperemic myocardial resistance is the dominant influence on vFFR and not the geometry of the lesion itself.

## Visual Abstract

**Figure:**
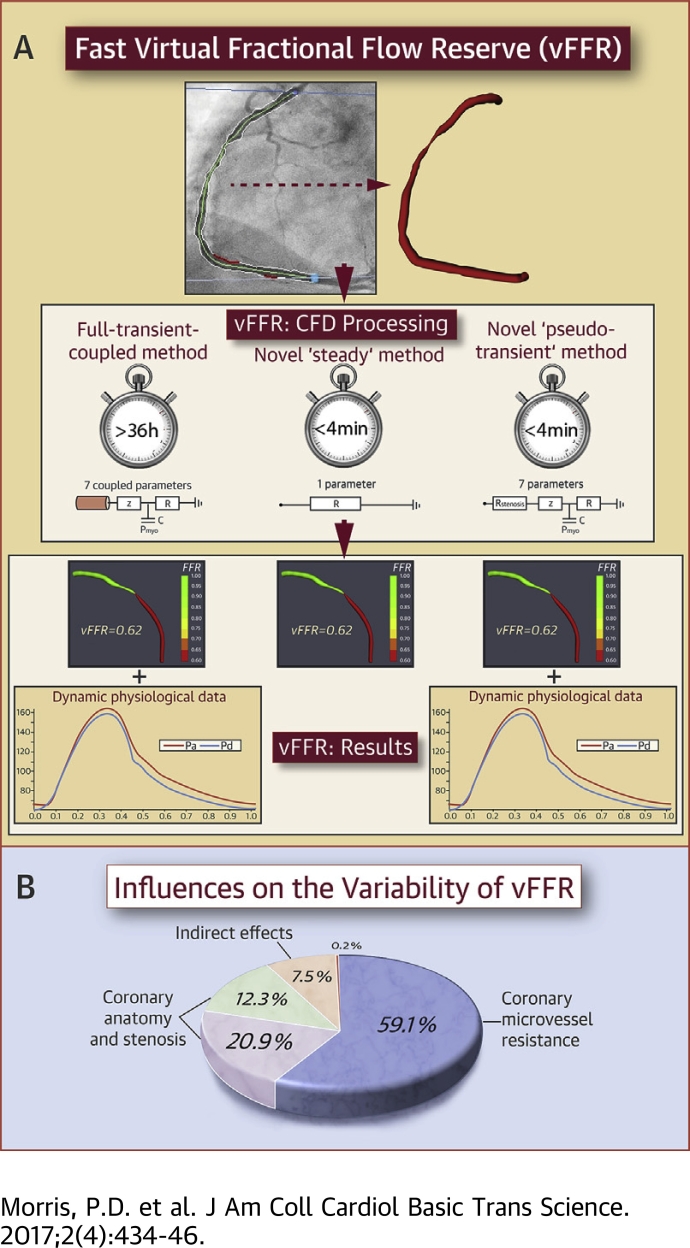

Fractional flow reserve (FFR) has become the standard of care for assessment of the physiological significance of coronary artery disease (CAD) 1, 2. When FFR is used to guide percutaneous coronary intervention (PCI), clinical outcomes are improved, fewer stents are deployed, and costs are reduced 3, 4, 5. However, even in countries where FFR is most frequently used, FFR is used in < 10% of PCI procedures and far fewer diagnostic cases 6, 7. This is due to a combination of factors related to practicality, time, and cost. Using computational fluid dynamics (CFD) to compute a “virtual” FFR (vFFR) from the coronary angiogram (CAG) is a way of making coronary physiology available to many more patients. vFFR can assess lesion significance without the insertion of a pressure-sensitive wire and without the induction of hyperemia. vFFR therefore offers the benefits of physiologically guided PCI without the drawbacks which limit the invasive technique. Although early results have been promising, 2 fundamental problems currently limit the usefulness of vFFR. The first problem is the time required to generate a result, which can be in excess of 24 h, due to the complexity of CFD solutions. Second, the precision of vFFR computation is limited by the accuracy by which the model represents the coronary and lesion geometry (imaging and reconstruction) and the physiological parameters (boundary condition tuning) on an individual patient basis (8). The current study resolves the former and sheds new light on the latter.

Our group has previously described a CFD-based method for computing vFFR from invasive CAG with good diagnostic accuracy (97%) (9). This method incorporated fully transient, 3-dimensional (3D) CFD analysis which requires substantial computing resources and typically took >24 h to produce a result. This approach is not appropriate for use in the cardiac catheter laboratory where on-table results are desirable.

Coronary blood flow follows a well-known pulsatile pattern. Modeling time-varying pulsatility requires “transient” (time-dependent) CFD analysis. However, FFR is calculated from mean pressure (and, by inference, flow) differences over time. This study hypothesized that complex transient CFD analysis might not be necessary. Steady-state CFD analysis runs several orders of magnitude more quickly than transient CFD analysis. However, it has yet to be determined whether an adequate estimation of the transient pressure and flow distributions could be made based on steady-state analysis results, particularly in the context of vFFR estimation. Predicting pulsatile vascular physiology on the basis of steady flow assumptions is not without precedent. In 1951, Gorlin and Gorlin (10) validated an equation which predicted cardiovascular orifice area from mean (steady) flow and disregarded flow pulsatility. More recently, both Tu et al. and Papafaklis et al. described systems capable of estimating coronary physiology, which are based upon steady-state CFD analysis 11, 12, 13.

The outputs of any model are determined by variations in input parameters which may occur due to natural biological variability or error in measurement. In the context of vFFR, these errors include a variety of geometric and physiological parameters. Promising vFFR results have been produced despite limitations in coronary imaging and segmentation and in the ways in which physiological parameters are used in model tuning 9, 14. It is important to understand the relative sensitivity of computed FFR to individual model input parameters. Sensitivity analysis is a formal mathematical process which allows the influence and interdependencies of individual model inputs to be decomposed and quantified in terms of their effects on model outputs, which in this case is the vFFR result.

The aims of the current study were first, to develop and validate a method which accelerated the computation of vFFR to a point which made it practical for use in the cardiac catheter laboratory; and second, to quantify the principal, accuracy-defining model features and parameters.

## Methods

### Study design

This was an observational, analytical, single-center study in which a novel “pseudotransient” analysis protocol for computing vFFR was developed and validated relative to both invasive FFR measurement and fully transient CFD analysis. All work was approved by the local ethics committee, and all participating patients gave informed consent.

### Patients

Patients were eligible for recruitment if they had proven CAD and were awaiting assessment for elective PCI. Apart from chronic total occlusion, all patterns and severity levels of stable CAD were eligible for recruitment. Exclusion criteria were acute presentation within 60 days; intolerance to intravenous nitrate, adenosine, or iodine-based contrast medium; coronary artery bypass graft surgery; or obesity which precluded CAG. Ethical approval and formal patient consent were obtained.

### Clinical protocol

Rotational coronary angiography (RoCA) was performed after isocentering in posterior-anterior and lateral planes after administration of glyceryl trinitrate, during a breath hold, with a hand injection of 10 to 20 ml of contrast. Fractional flow reserve was measured in the standard way (15), across all lesions with >50% vessel diameter by visual estimation, under baseline and hyperemic conditions, using intravenous adenosine, 140 μg/kg/min (Volcano Corp., San Diego, California) with the pressure transducer positioned at least 4 reference vessel diameters distal to the end of the lesion. Percutaneous coronary intervention proceeded according to the operator’s normal practice, guided by the angiogram and the measured FFR. To ensure a diverse and wide-ranging case mixture, RoCA and physiological measurements were repeated post PCI and under baseline and hyperemic conditions.

### Segmentation and meshing

**Figure 1:**
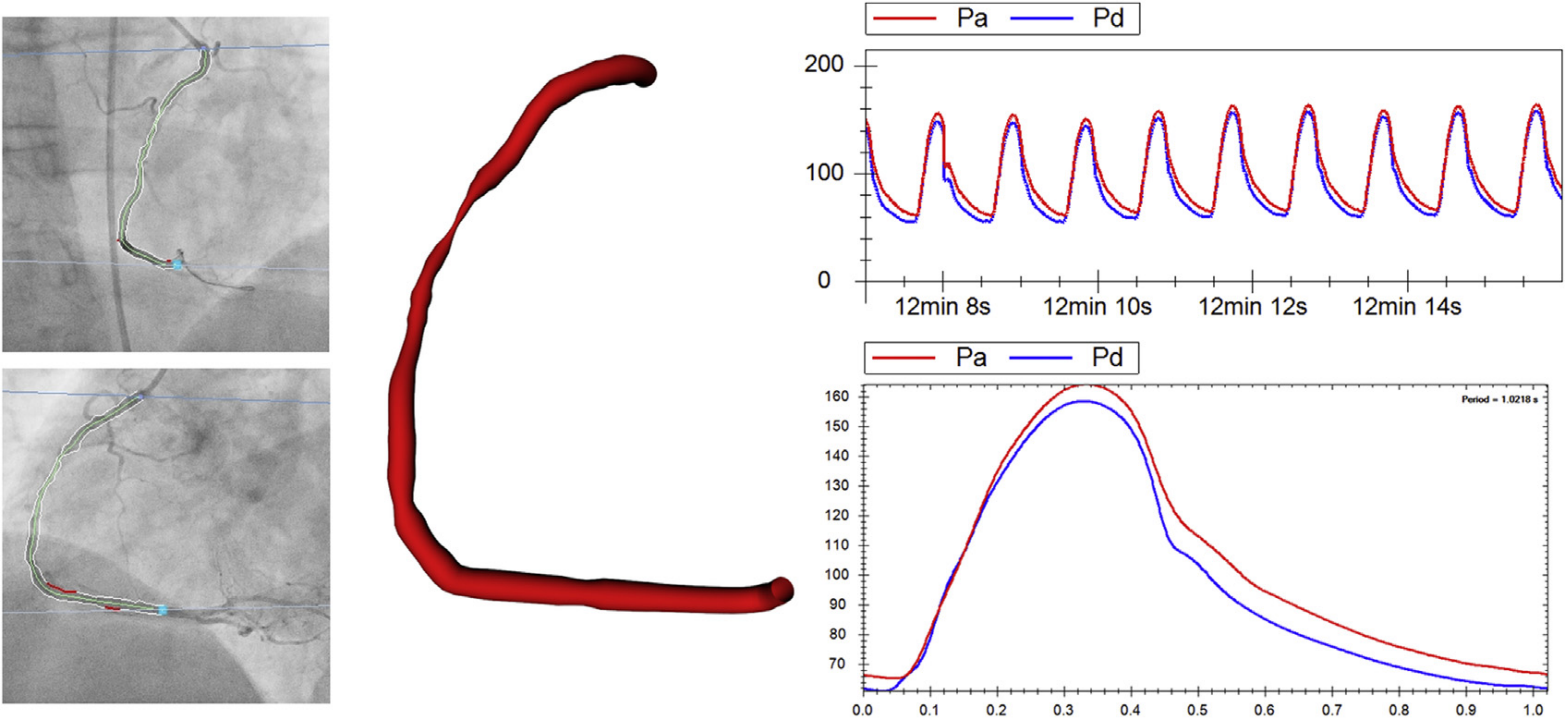
Processing Raw Clinical Data Angiograms of a diseased right coronary artery **(left)** have been segmented, and the reconstructed vessel is shown **(middle)** alongside the processed pressure data **(right)** within the VIRTUheart workflow environment.

**Figure 2:**
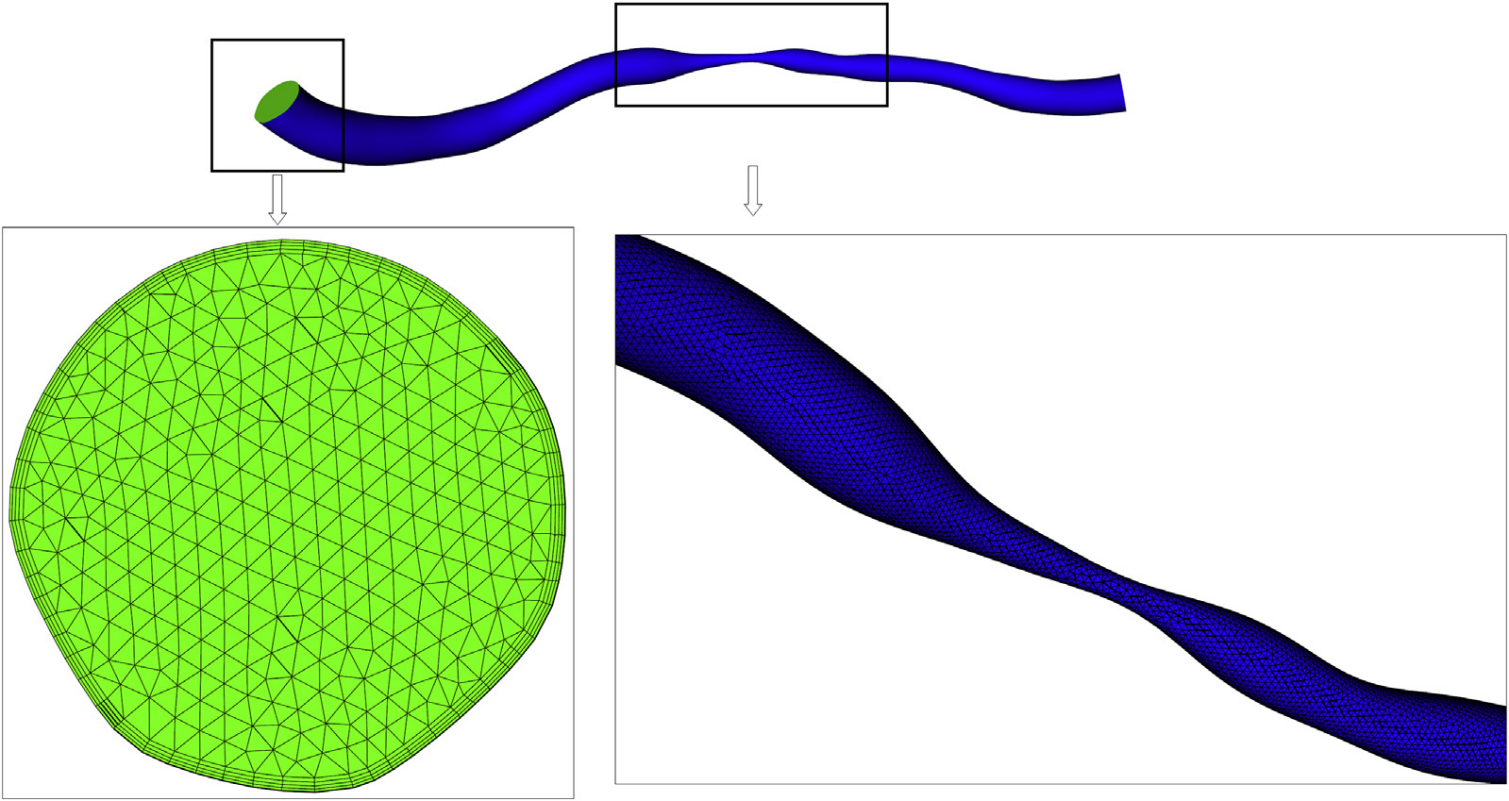
Sample Finite Element Mesh Used for Simulations Mesh shown is produced from the angiogram shown in Figure 1. Details of the wall **(blue)** and inlet **(green)** are shown. The near-wall region is refined using prism elements.

Vessels were reconstructed from the angiogram images (Allura 3D-RA system, Philips Healthcare, Best, the Netherlands) by a cardiologist experienced in such methods (Figure 1). A 1- to 2-million element volumetric mesh was fabricated in ICEM (ANSYS, Canonsburg, Pennsylvania) for each unique arterial geometry (Figure 2). Depending on the size of the segmented vessel segment, the volumetric meshing process took between 1 and 4 min. Computation and all simulations were performed using ANSYS CFX (ANSYS, Inc., Cannonsburg, Pennsylvania) on a Precision T5600 computer (Intel Xeon processor, 32GB RAM, Dell, Round Rock, Texas).

### Fully transient vFFR model

Our gold standard reference method was vFFR computed by fully transient 3D CFD. It was computed in a method consistent with the methods described in the VIRTU-1 (VIRTUal Fractional Flow Reserve From Coronary Angiography) trial (9). Catheter pressure was applied at the proximal boundary. The distal boundary of the 3D domain was coupled to a 7-element, modified Windkessel model, applying the coronary microvasculature (CMV) parameters derived by the optimization process described below. The pressures were uniform at the boundaries, and no velocity profiles were imposed.

### Pseudotransient vFFR analysis protocol

A pseudotransient vFFR protocol (virtual fractional flow reserve computed with the pseudotransient steady-state method [vFFR_ps-trns_]) was developed so that transient results could be approximated without performing fully transient CFD analysis. A transient analysis of a 2-compartment model, consisting of a compartment representing the diseased arterial segment and a compartment representing the distal microvasculature, was performed. The proximal compartment was represented by a quadratic equation relating the instantaneous pressure drop to the instantaneous flow. The coefficients of this equation, z_1_ and z_2_, for the linear and quadratic terms, respectively, were determined from 2 steady-state 3D CFD analyses of the arterial geometry with prescribed flow rates, with a plug velocity profile and a uniform zero-pressure outlet (see Supplemental Appendix A for derivation). A range spanning sub- to supraphysiological flow rates was evaluated (0.5, 1, 2, 3, 4, 5 ml/s) in order to identify the 2 flow rates which optimally characterized the terms z_1_ and z_2_ over the cohort investigated. vFFR_ps-trns_ was a function of 9 parameters: the proximal pressure trace, terms z_1_ and z_2_, CMV resistance, CMV compliance, and the parameters describing the myocardial systolic contraction including pressure generation, plateau, decay, and amplitude (see Supplemental Appendix B for the derivation of vFFR_ps-trns_).

### Steady vFFR model

**Figure 3:**
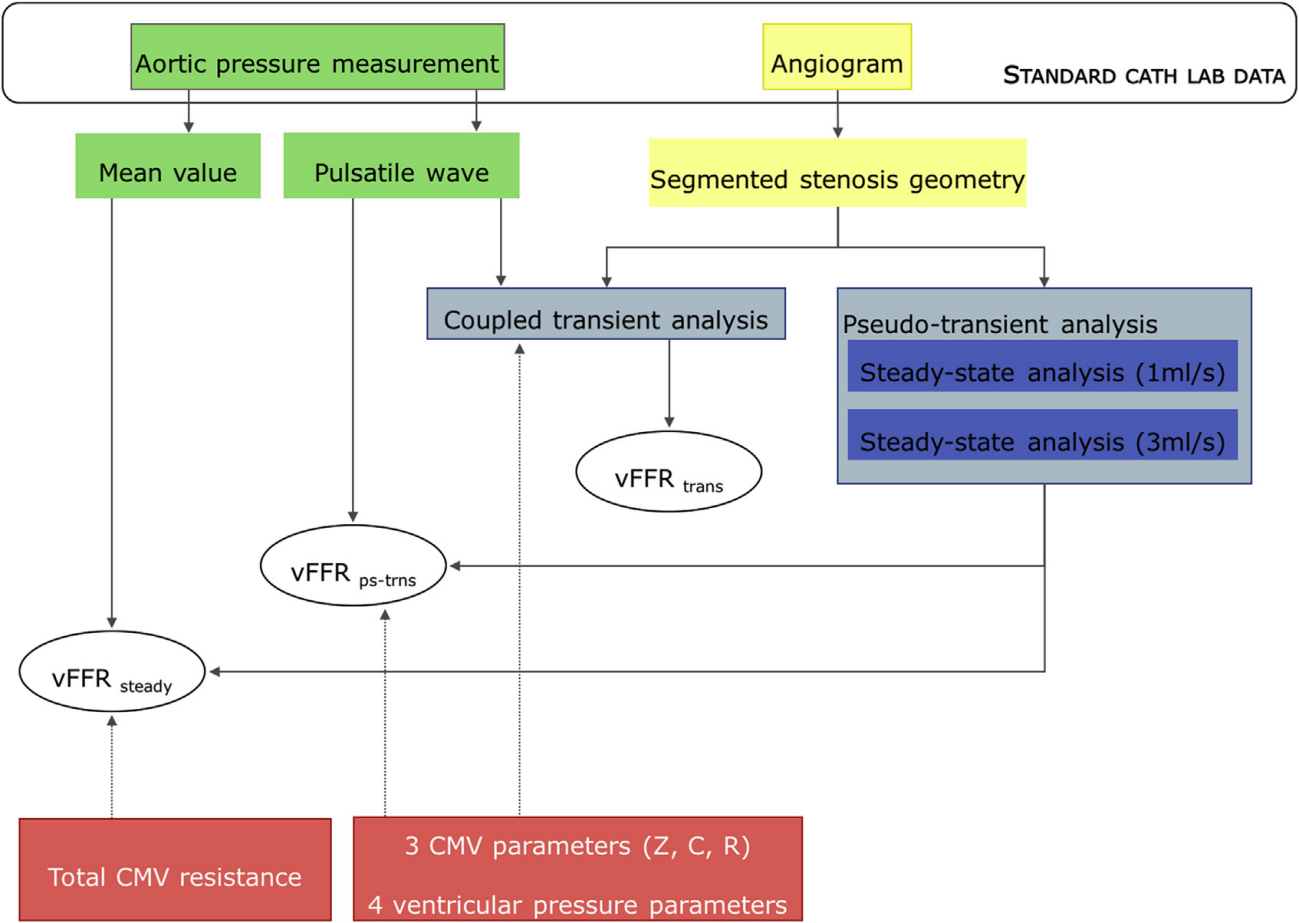
Models for Computing vFFR The imaging and pressure input data for both novel models are those collected during routine coronary angiography (image data in **yellow** and aortic pressure data in **green**). The parameters of CMV physiology must be estimated **(red)**. The type of simulation used to calculate vFFR values are shown in the **blue boxes**. vFFR_ps-trns_ is a function of 9 parameters, whereas vFFR_steady_ is a function of 4. Pseudotransient flow can be reconstructed using a 1D flow model representing the 3D vessel geometry coupled to the 0-dimensional Windkessel model. C = compliance; CMV = coronary microvasculature; R = resistance; vFFR = virtual fractional flow reserve; Z = impedance.

The pseudotransient analysis protocol computed an approximation of the temporal variation of pressure distal to the lesion from which, together with the proximal pressure, the vFFR was calculated. The transient waveforms have value beyond the computation of vFFR, but given that FFR is a measurement computed from average pressure, it was interesting also to investigate whether vFFR can be computed from a single steady flow analysis at the mean pressure. The protocol was further simplified to calculate “steady” vFFR (vFFR_steady_). In the computation of vFFR_steady_, all distal parameters of CMV physiology were reduced to a single, time-averaged value of resistance. vFFR_steady_ was therefore a function of just 4 parameters: mean proximal pressure, terms z_1_ and z_2_, and total distal resistance. This method does not require (or allow) accurate reconstruction of the aortic pressure wave because the mean pressure is used. The major advantage of this method is that it requires far fewer parameters; however, there is little advantage in computational speed over the pseudotransient analysis. Derivation of the equation for calculating vFFR_steady_ is described in Supplemental Appendix C. A comparison of the data used in each model is demonstrated in Figure 3.

### Boundary data

The proximal physiological boundary condition was the patient-specific pressure measured from the guiding catheter which sits at the coronary ostium (i.e., aortic pressure). The distal compartment requires a characterization of distal impedance. This was computed for each individual from the invasive pressure measurements at the proximal and distal boundaries by using the guiding catheter and pressure wire (Volcano Corp.), respectively. The outlet of the reconstructed vessel corresponded to the location of the pressure wire transducer, the location of which was specifically recorded during CAG. This is consistent with standard FFR measurement. Because the distal boundary condition was based upon measured values, the computed vFFR was expected to be accurate. The focus of this study was not a clinical trial of vFFR but rather an analysis of the speed and accuracy of the novel CFD acceleration techniques described below. The challenge of tuning the parameters of the distal impedance model to the individual is discussed in detail elsewhere (8). The important result in this study is, therefore, not whether vFFR matches mFFR but rather, can the accelerated methods generate results which do not sacrifice accuracy relative to the fully transient 3D analysis. A semiautomatic optimization algorithm was developed within Matlab (Mathworks Inc., Natick, Massachusetts) to derive the parameters of the CMV from invasively measured values. This method was based on the method by Lungu et al. (16), who provided a full description. These parameters included CMV impedance, resistance, and compliance, along with 4 parameters reflecting the amplitude and timing of intramyocardial systolic pressure.

### Sensitivity analysis

A formal sensitivity analysis was performed using the Sobol decomposition method 17, 18. The input parameters examined were CMV resistance, geometry parameters (z_1_ and z_2_), and proximal pressure (P_a_). This variation-based method can be used to determine the magnitude of effect of individual input parameters on the output of a model, in this case vFFR. The main sensitivity indices provide information about the reduction in model output variation if an input factor would be accurately applied. Therefore, the main sensitivity indices can provide a ranking of the individual input parameters responsible for proportions of the model output variation (i.e., the inputs are ranked in order of the level of influence on the vFFR result that they have). Additionally, total sensitivity indices can be used to identify parameters that have little or even negligible effect on model output and can therefore be fixed to population averages. For the current study, the main and total sensitivity indices were determined over parameter ranges derived from the patient cohort by using the vFFR_steady_ method. Definitions of the sensitivity indices can be found in Supplemental Appendix D.

### Statistical analysis

The diagnostic accuracy of the workflow (ability of vFFR to predict FFR <0.80 or >0.80) was assessed by calculating the sensitivity, specificity, positive predictive value, and negative predictive value. Overall accuracy was calculated as the true positive-to-true negative results ratio to the total number of cases. Agreeability between vFFR and FFR was assessed by calculating the bias, mean delta, and standard deviation of the mean delta and was demonstrated graphically as a Bland-Altman plot (19). Time-dependent error between transient and pseudotransient results is expressed as the normalized root mean square (RMS) norm (i.e., the measured transient distal pressure vector is subtracted from the computed pseudotransient one, normalizing by dividing by the mean transient pressure and computing the 2-norm [RMS]) of this vector. Because FFR is calculated using cycle mean values, the error between the means is also expressed. The intraclass correlation coefficient is reported as a measurement of agreeability between the vFFR metrics. Unless stated otherwise, data are mean ± SD. Comparison of means was performed using a Mann-Whitney *U* test. Statistical analysis was performed using SPSS Statistics version 22 (IBM Analytics, Armonk, New York).

## Results

### Patient and clinical characteristics

Data were collected from 20 patients. Their baseline characteristics are summarized in Table 1. In total, 73 unique arterial datasets were studied (Table 2), which consisted of 34 left anterior descending, 21 right coronary, 3 diagonal, 7 left circumflex, and 8 left main coronary arteries. A total of 39 cases were pre PCI, 25 cases were post PCI, and 9 cases did not receive a stent; 41 cases were under hyperemic conditions, and 32 cases were baseline measurements. Mean SYNTAX (Synergy between PCI with TAXUS drug-eluting stent and Cardiac Surgery) score was 10.45 (20), and mean New York PCI Risk Score was 0.22 (21).

**Table 1:** Baseline Characteristics

Baseline characteristics	
Age, yrs	66 (51–87)
Male	70%
Body mass index, kg/m^2^	29.6 (3.4)
Comorbidities	
Hypertension	60%
Hyperlipidemia	90%
Diabetes	30%
Current smoker	0%
Prior myocardial infarction	45%
Stroke	0%
Peripheral vascular disease	15%
Medication	
Aspirin	90%
Beta-blocker	65%
Nitrate	60%
Statins	90%
ACE inhibitors	45%
Calcium-channel blockers	25%
Clopidogrel	75%
ARBs	20%

Values are mean (range), %, or mean (%).

ACE = angiotensin-converting enzyme; ARB = angiotensin receptor blocker.

**Table 2:** Comparison Among Pseudotransient and Steady vFFR Methods Relative to Measured Translesional Pressure Ratio Values

	N	Error	Bias	Max Error Range∗
All cases				
Pseudotransient	73	0.0070 ± 0.0045	−0.0051 ± 0.0065	−0.018 to +0.013
Steady	73	0.0044 ± 0.0044	−6e^−4^ ± 0.0062	−0.011 to +0.022
FFR <0.90				
Pseudotransient	37	0.0094 ± 0.0038	−0.0080 ± 0.0063	−0.018 to +0.013
Steady	37	0.0050 ± 0.0049	−9.7e^−5^ ± 0.0070	−0.011 to +0.022
FFR 0.70–0.90				
Pseudotransient	29	0.0098 ± 0.0037	−0.0090 ± 0.055	−0.018 to +0.013
Steady	29	0.0048 ± 0.0045	−3.1e^−4^ ± 0.0067	−0.011 to +0.022

Values are mean ± SD unless otherwise indicated.

vFFR = virtual fractional flow reserve.

∗ Indicates worst underestimation to worst overestimation.

### Selection of steady flow rates for characterization

We performed 438 steady-state simulations (73 cases, each at 0.5, 1, 2, 3, 4, and 5 ml/s), and a quadratic pressure-drop versus flow relationship was computed from each pair of flow rates (Supplemental Appendix B). When we compared the errors between the steady-state method (pseudotransient) and measured FFR, the best overall accuracy was produced when z_1_ and z_2_ were derived from steady-state flows simulated at 0.5 and 5 ml/s (vFFR mean error vs. measured values: ±0.0043 [0.004]). However, neither of these flow rates are physiological in the context of stable CAD. Furthermore, at a flowrate of 5 ml/s, 1 steady-state analysis became unstable and failed to converge to a satisfactory result due to an excessively high Reynold’s number (i.e., unrealistic physics for biological flow through a tight stenosis). For this reason, flow rates of 1 and 3 ml/s were selected as the bases for the characterizations. This pairing is more representative of the prevailing underlying physiology, converged to a satisfactory result expediently in all cases, and was associated with an error which was only mildly higher than that of the 1 and 5 ml/s pairing (mean error: ±0.0069 [0.005]). The 1 and 3 ml/s pairing was therefore adopted for subsequent analysis.

### Steady-state CFD analysis time

Using the 1 and 3 ml/s flow pairings, all 73 steady-state flow pairings converged successfully at the first attempt with no alteration to the protocol. The mean total time for 1 pair of steady-state analyses (the basis for the pseudotransient and steady vFFR workflows) was 189.3 ± 34.0 s.

### Accuracy of pseudotransient vFFR

**Figure 4:**
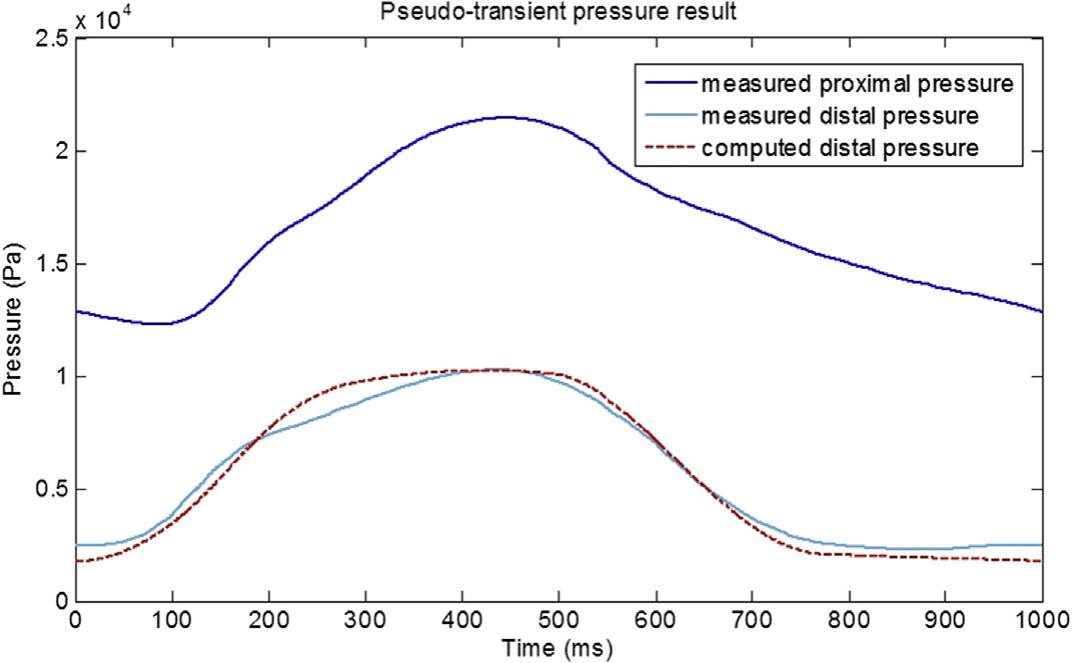
A Pseudotransient Pressure Result From an LAD Arterial Case Invasively measured FFR was 0.350 and the computed vFFR was 0.346. The pseudotransient result closely matches the invasively measured result (RMS norm: 0.026), despite no transient data being used in its computation. RMS = root mean square; other abbreviations as in Figure 3.

**Figure 5:**
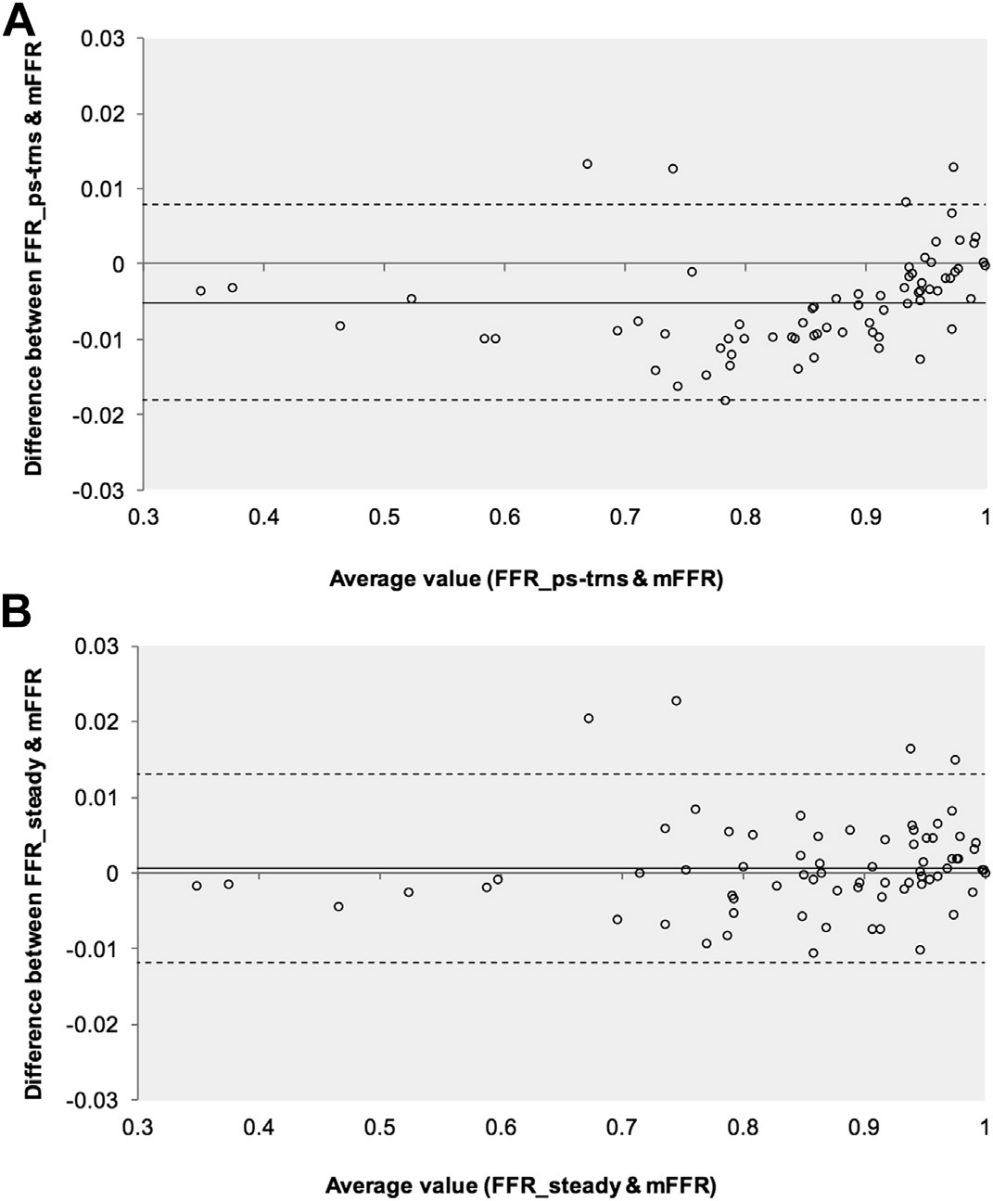
Bland-Altman Plots Demonstrating Agreement Between vFFR and Measured FFR The **solid line** indicates bias (mean delta), and the **interrupted lines** represent the upper and lower limits of agreement (SD: ±1.96). **(A)** Agreement for vFFR_ps-trns_, and **(B)** agreement for vFFR_steady_. Note the high number of cases in the clinically important FFR range from 0.7 to 0.9.

An example of a pseudotransient result plotted relative to measured data is demonstrated in Figure 4. The high accuracy compared with measured FFR was because the distal impedance was tuned using the (measured) distal pressure. The primary challenge in using the analysis protocol for accurate computation in the clinical setting to replace measured FFR is in the prior and independent estimation of the distal impedance (17), but the focus of this paper was to show that a computationally inexpensive analysis protocol based on steady flow analyses can replace a fully transient CFD study. A Bland-Altman plot is shown in Figure 5A. Agreement between vFFR_ps-trns_ and measured data was also high (Table 3). In percentage terms, relative to measured values, mean error was ±0.86% (0.60). The intraclass correlation coefficient between vFFR_ps-trns_ and mFFR was 0.999 (95% confidence interval [CI]: 0.998 to 0.999; p < 0.001). vFFR_ps-trns_ achieved 100% sensitivity, specificity, positive predictive accuracy, negative predictive accuracy, and overall diagnostic accuracy for diagnosing physiological lesion significance (FFR <0.80 or >0.80). Pseudotransient results were compared with those derived from values measured for goodness-of-fit. An example of a measured transient versus pseudotransient result is demonstrated in Figure 4. Over all 73 datasets, the RMS norm between the pseudotransient results and measured data was 0.37 ± 0.49. However, this was significantly better in the cases where FFR was <0.90 (RMS norm 0.15 ± 0.34).

**Table 3:** Effect of Applying Generic (Averaged) Boundary Conditions on Quantitative and Diagnostic Errors

Basis Upon Which Subgroupings Were Averaged (CMVR_total_)	N Datasets	Error of vFFR Result	Bias (Mean Delta)	Diagnostic Accuracy
All cases	73	0.11 ± 0.12	0.11 ± 0.12	75%
Baseline and hyperemic conditions	73	0.096 ± 0.096	0.088 ± 0.104	52.1%
Right and left coronary arteries (under hyperemic conditions)	40	0.078 ± 0.079	0.046 ± 0.102	80%
Artery-specific (LAD, RCA, DX, LMCA, LCX) (under hyperemic conditions)	40	0.0050 ± 0.0046	−2.6 e^−5^ ± 0.0068	82%
Case-specific (no averaging)	73	0.0044 ± 0.0044	−6.0 e^−4^ ± 0.0062	100%

Values are mean ± SD unless otherwise indicated.

CMVR_total_ = total coronary microvascular resistance; DX = diagonal artery; LAD = left anterior descending artery; LCX = left circumflex artery; LMS = left main coronary artery; RCA = right coronary artery; other abbreviations as in Table 2.

### Accuracy of vFFR_steady_

Agreement between vFFR_steady_ and measured FFR was also high (Table 2). A Bland-Altman plot is shown in Figure 5B. In percentage terms, relative to measured values, mean error was ±0.50% (0.40). The intraclass correlation coefficient between vFFR_steady_ and mFFR was 0.999 (95% CI: 0.998 to 0.999; p < 0.001). vFFR_steady_ also achieved 100% sensitivity, specificity, positive predictive accuracy, negative predictive accuracy, and overall diagnostic accuracy when we diagnosed physiological lesion significance (FFR ≤0.80 or >0.80).

### Comparison with transient results

For comparison, the complex, zero-dimension–coupled, fully transient method was compared with the vFFR_ps-trns_ and vFFR_steady_ methods. The mean time for the completion of the fully transient CFD analyses was 26 h,48 min (range: 6 to 48 h). The steady-state method therefore was processed more than 500 times faster than the fully transient analysis. Unlike the steady-state analyses, the transient CFD analyses became repeatedly unstable, necessitating reductions in the simulation time-step (at the expense of increasing computation time). Mean error for the transient method (±1.0%) was not statistically significantly different from vFFR_ps-trns_ and vFFR_steady_ methods in a small subset of 6 transient cases.

### Areas of clinical interest

According to published studies, the ischemic threshold corresponds to an FFR of ≤0.80. FFR is typically used to help determine the best course of action in angiographically significant or borderline cases. Accuracy of both of the steady-state vFFR methods was therefore also assessed in subgroups of cases where FFR was <0.90 (n = 37) and more borderline cases where FFR was 0.70 to 0.90 (n = 29) (Table 2). There were no statistically significant differences in accuracy of either method when deployed in either subgroup or when deployed in all cases (Table 2).

### Sensitivity analysis

**Figure 6:**
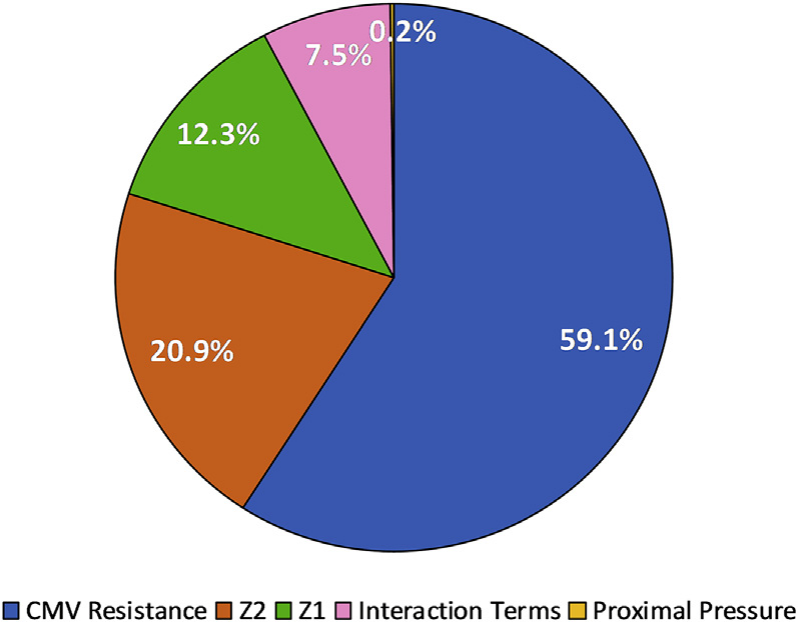
Pie Chart Demonstrates Relative Effect of Each Individual Model Input Parameter on Model Output (vFFR) Variance See Supplemental Appendix D.

**Figure 7:**
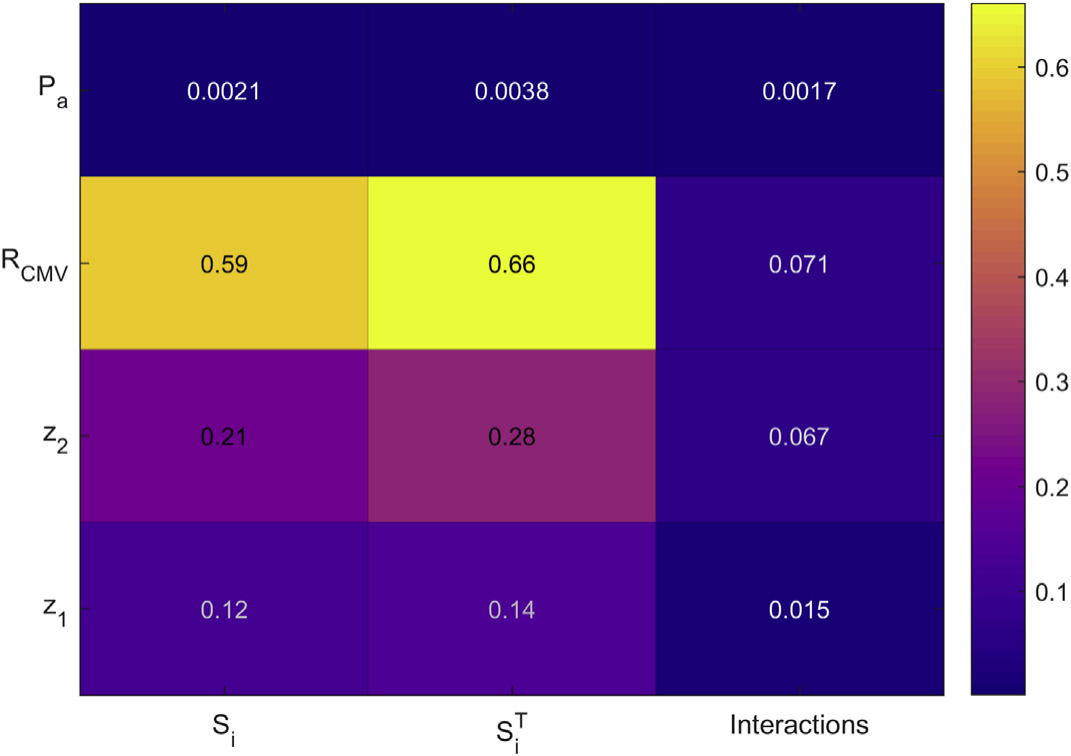
Sensitivity Index Heatmap The main sensitivity indices (S_i_), total sensitivity indices (S_i_^T^), and interaction effects are displayed for the 4 input parameters: R_CMV_, geometry parameters (z_1_ and z_2_), and average proximal pressure (P_a_). The axis on the **right** indicates the magnitude of the influence on output (vFFR) result, with higher values having a stronger influence on result. CMV resistance is the dominant influence on vFFR result. CMV = coronary microvascular; R_CMV_ = CMV resistance; other abbreviations as in Figure 3.

**Figure 8:**
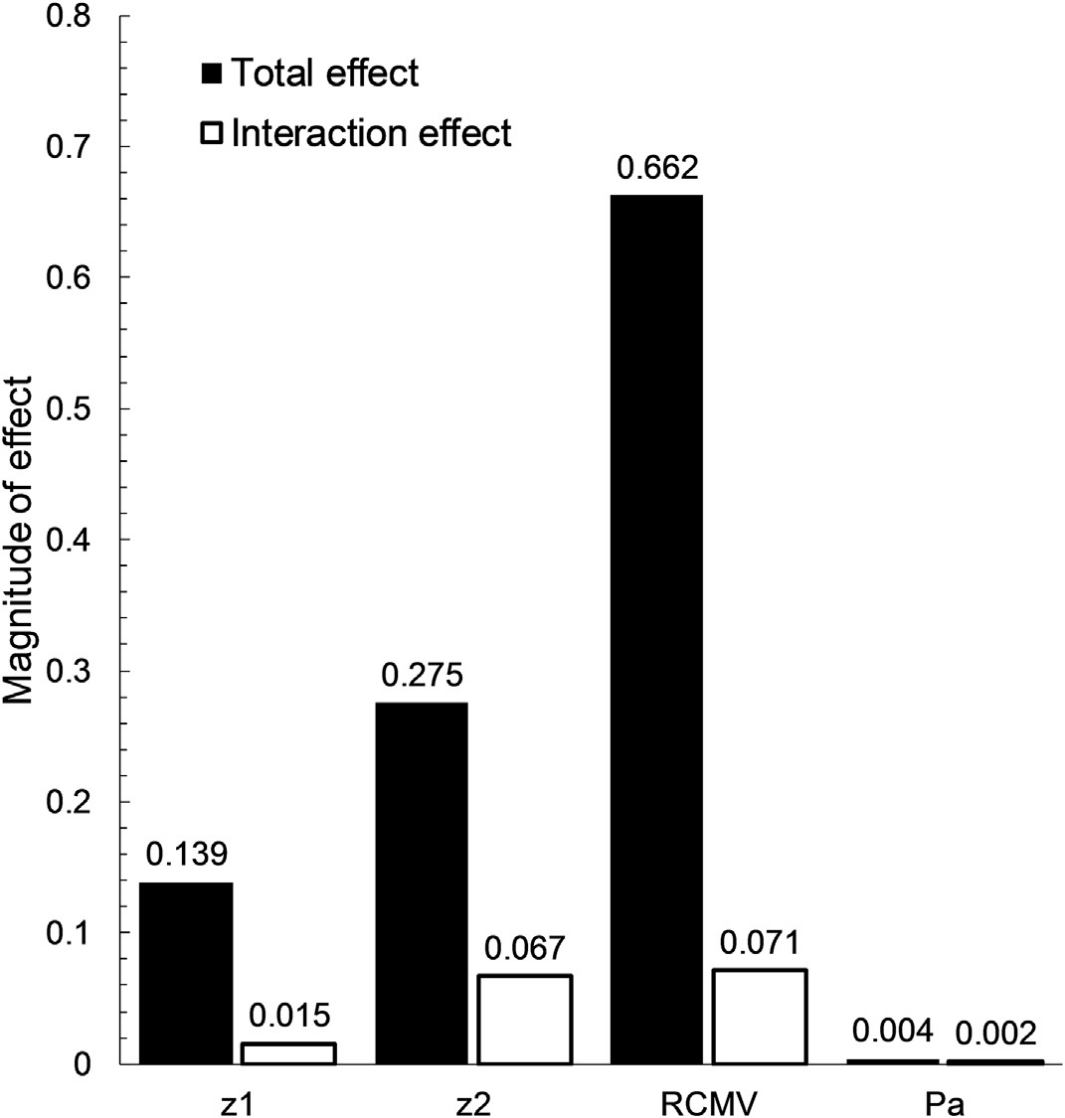
Total and Interaction Model Input Effects Bar chart demonstrating the magnitude of the total (direct and interactions) effect on vFFR caused by the input parameters CMV resistance (RCMV), geometry parameters (z_1_ and z_2_) and average proximal pressure (Pa). Abbreviations as in Figure 7.

Figure 6 provides a ranking of the main sensitivity indices and demonstrates that the principal influence on the variation of vFFR values was the total distal CMV resistance, accounting for 59.1% of the variation. Coronary anatomy and stenosis geometry (characterized by z_1_ and z_2_) were of secondary importance in the study population, accounting for 33.2% of vFFR variation. A heatmap of the FFR sensitivity indices is displayed in Figure 7. Only 7.5% of the model output variation was caused by higher order interaction effects. Interaction (indirect) effects are defined as the difference between the total effect and the direct (main) effect of an input parameter. The magnitude of the interaction effects is demonstrated in Figure 8, which displays the total sensitivity indices divided into the main effects (S_i_) and the remainder accounting for higher-order interaction effects. A relatively small proportion of the total effect of the individual input parameters can be attributed to interaction effects. The total variation in vFFR caused by proximal pressure (P_a_) is <1%, as demonstrated by the total sensitivity index value of 0.0038. Therefore, average proximal pressure had negligible effect upon vFFR result.

### Generic boundary conditions

The influence of CMV resistance was further demonstrated by reanalyzing all cases, applying a generic value of CMV resistance as the distal boundary condition. This universal value was the mean resistance from all included cases. The effect this had upon vFFR error is demonstrated in Table 3. Accuracy improved as the averaged value for CMV applied at the distal boundary better and more specifically reflected the coronary arterial subgrouping.

## Discussion

We have developed a pseudotransient analysis protocol for the fast and accurate computation of vFFR. It requires 2 steady-state CFD analyses to derive the linear and quadratic terms which characterize the relationship between pressure and flow for each unique arterial case. The computational execution time is approximately 3 min on a standard desktop PC, and given an accurate measure of distal impedance, the protocol quantified FFR with <1% error and was 100% accurate in diagnosing physiological lesion significance, relative to the gold standard reference of fully transient CFD analysis. An even simpler, fully steady protocol also gave similar results for FFR but at almost the same computational expense and without the benefit of representation of the transient waveforms. These results suggest that time-consuming, high-powered computer processing for complex transient CFD analysis is not necessary for the computation of vFFR. Furthermore, steady-state analysis was more robust and reliable than fully transient analysis, which proved unstable in a number of cases. We applied a rigid wall simulation which simplified the CFD solution. Although this disregards coronary vascular compliance, this approach has previously been demonstrated to be acceptable in coronary hemodynamic modeling (22) and appears appropriate in the current study.

The pseudotransient protocol runs in 0.2% of the time taken for fully transient CFD analysis. The magnitude of this acceleration means it is now feasible that vFFR can be computed in less time than it takes to measure FFR invasively with a pressure-sensitive wire. Computational fluid dynamics analysis is now no longer the rate-limiting step in vFFR computation. Instead, segmentation, meshing, and pre-processing protocols are now the time-critical processes. At the current stage of development, these additional components of the workflow (which are not the focus of this study) run in approximately 2 to 4, 2, and 2 min, respectively.

Aside from system acceleration, the sensitivity analysis performed in this study has demonstrated the relative importance of each input parameter in the precision of vFFR computation. The fact that vFFR was insensitive to variations in P_a_ is consistent with the original clinical FFR studies by Pijls et al. (15) and De Bruyne et al. (23). For many years, angiographic assessment of CAD focused solely upon lesion anatomy. More recently, it has been demonstrated that physiological assessment (e.g., FFR) correlates more closely with clinical outcome than lesion anatomy alone. Several groups have tried, largely unsuccessfully, to infer physiological lesion significance purely from lesion anatomy by using 2D and 3D quantitative coronary angiography (QCA) 24, 25, 26. The sensitivity analysis presented in this study explains this collective failure: the aforementioned studies did not incorporate any measurement of CMV resistance in their processing. This study of a population of patients with stable coronary artery disease demonstrates that variability in CMV resistance had a greater influence on the vFFR result than variability in epicardial coronary and lesion anatomy. This highlights how critical it is to include an accurate estimate of CMV resistance into any virtual FFR model. This also explains the strength and success of FFR over standard and quantitative CAG, because invasive FFR measurement automatically incorporates the magnitude of CMV resistance (15). It also explains why other published work in this area has been able to report reasonable vFFR accuracy, despite using relatively imprecise imaging and segmentation protocols 9, 12, 14. If modelers wish to construct workflows which accurately compute FFR, without wire insertion and without the induction of hyperemia, tuning the model parameters of CMV resistance on an individual case basis now represents the single greatest challenge to overcome (17). The application of completely generic boundary conditions yielded inferior accuracy. When we applied subcategorized averages based upon arterial subtype, accuracy improved, supporting the notion that the precision of the CMV resistance is critical to computing physiological lesion significance.

The ability to predict transient values without performing transient analysis is also a significant development. The quality of the fit between pseudotransient and actual values was dependent upon the accuracy of the Windkessel parameters applied (CMV resistance, impedance, compliance and intramyocardial systolic pressure). Although the accuracy of both of the novel methods was high across a wide range of FFR values tested (Table 2), for methodological reasons, parameter derivation is improved when the translesional pressure gradient is greater. This explains why accuracy appears to be slightly improved for cases where the FFR was lower (Figure 5). Not only is this effect subtle, it is not related to the accuracy of the computational model but, separately, of the parameterization strategy used. The accuracy and value of the pseudotransient method are impressive and applicable to a wide range of engineering applications (along with but not necessarily limited to models with scales of similar length and Reynold’s numbers) beyond the cardiovascular system and even beyond biological modeling. The pseudotransient method retains the high temporal and spatial resolution of the transient analysis but relies upon faster and more robust steady-state analysis. In the current study, the Navier-Stokes equations of fluid flow were solved using CFX (ANSYS). The drawback of the pseudotransient method is that, in addition to z_1_ and z_2_, it requires the application of 7 parameters (impedance, resistance, compliance, and 4 parameters describing the timing and amplitude of intramural myocardial systolic pressure), whereas the steady method only requires resistance (Figure 3). This may be simple in nonbiological modeling, but deriving these parameters noninvasively remains a significant challenge for the current application.

The accuracy of the steady analysis protocol (vFFR_steady_) demonstrates how simple the computation of vFFR can be. This protocol requires only the vessel geometry (to derive z_1_ and z_2_) and the total distal resistance of the CMV (Figure 3).

Apart from lesions causing chronic total obstruction, all patterns and severities of CAD, including left main coronary artery disease were included in this study. The current study therefore reflects “real world” working practice and is widely applicable. A further strength of the current methods is that they can be applied to any arterial geometric reconstruction including CAG and coronary computed tomography angiography (CCTA).

Other authors have used steady-state analysis in a similar context. Papafaklis et al. (12) used paired steady-state analysis to compute “virtual functional assessment index” (vFAI) for fast functional assessment of intermediate coronary lesions, which was also based upon steady-state analysis. vFAI was computed in 7 min, based on the distal pressure-to-proximal pressure ratios over the lesion for flows in the range of 0 to 4 ml/s, normalized by the ratio over this range for a normal artery. vFAI is numerically equal to the average of the computed pressure ratio over this flow range. Although this is likely to be superior to QCA-derived functional lesion assessment (because the geometric description is transformed into a more physiologically relevant measurement, namely pressure ratio, by the computation of the relationship between pressure ratio and flow), vFAI is entirely a function of the geometry of the stenosis. The results of the current sensitivity analysis demonstrated the critical importance of distal resistance. Because vFAI ignores this parameter, it cannot be a surrogate for FFR. vFAI will indicate the need for intervention if the lesion is geometrically significant, whereas the FFR might be high or low for the same lesion depending on the overall physiology and, particularly, on the status of the coronary microvasculature.

Tu et al. (11) developed a 3D steady-state model to predict vFFR. Arterial segmentation was from CAG images. Computational fluid dynamic simulation was completed in approximately 5 min. Mean hyperemic coronary flow was estimated from Thrombolysis In Myocardial Infarction (TIMI) frame counting of the rapidity of the contrast wave front within the coronary arteries during injection. This approach is advantageous because it can be estimated during routine angiography and is computed in a similar time scale. However, a disadvantage is that it requires induction of hyperemia (another potential factor contributing to FFR underuse); and because it is only an estimation of mean flow, the method cannot generate transient (or pseudotransient data). More recently, Tu et al. (11) described a method for computing quantitative flow ratio (QFR). Not only is QFR quick to compute but tuning can be performed based upon contrast flow (cQFR) without inducing hyperemia. Compared with mFFR, QFR identified physiological lesion significance with 86% overall accuracy (13).

### Study limitations

First and most importantly, in this study, we inferred the parameters of the CMV from invasive measurement. In a truly predictive study, where invasive measurement is avoided, these would not be known. However, the aim of this study was to develop and validate a method of vFFR computation which could operate within time scales which are practical for real-time use in the cardiac catheter laboratory. Tuning the boundary conditions to reflect CMV physiology remains a challenge for all groups working in this area and represents a work in progress for the study team. Second, the simplified approach applied in this study ignored the influence of side branches proximal and distal to a lesion. A schema for distributing pressure and flow among branches is currently under development. Third, the image segmentation protocol used is based upon RoCA, which is not widely available and produces axis-symmetrical coronary lumen segmentations. However, this study demonstrates that when vFFR is computed, geometric precision is of secondary importance to the precision of the CMV resistance. Furthermore, the methods developed in this study are applicable to any coronary segmentation. Fourth, the current sensitivity analysis examines the sensitivity of the model to interpatient variability (leading to variability in vFFR prediction). The variation of all vFFR values has been decomposed and attributed to the individual model parameters (or combinations of these parameters). This study did not address intrapatient sensitivity and uncertainty of vFFR predictions due to measurement uncertainties. This is something we intend to develop for future iterations of the vFFR workflow and will be formally examined on a patient-by-patient basis.

## Conclusions

Given an accurate value for CMV resistance, vFFR can be accurately computed from CAG in <4 min, without the insertion of a pressure wire and without induction of hyperemia. Transient results can be predicted without performing time-consuming transient CFD analysis. Accuracy of vFFR computation is influenced less by geometric accuracy and more upon the tuning of the model to accurately represent distal CMV resistance.

Perspectives **COMPETENCY IN MEDICAL KNOWLEDGE:** The gold-standard assessment of physiological lesion significance is the invasive measurement of FFR, but clinical uptake remains low. Investigators have attempted to compute vFFR by using CFD modeling to provide the benefits of physiological lesion assessment without the drawbacks limiting the invasive method. The novel mathematical methods described in this article reduce vFFR computation time from >36 h to <4 min on a standard desktop computer, without compromising clinical accuracy or transient (time-dependent) physiology. Sensitivity analysis demonstrates that myocardial resistance, not lesion geometry, is the dominant influence on vFFR results.

**TRANSLATIONAL OUTLOOK:** The final major barrier to a reliable vFFR tool is the application of a patient-specific tuning strategy to represent either hyperemic flow or myocardial resistance.

## Appendix

Supplemental Appendices A–D
