# Supplementary material for: Fast Virtual Fractional Flow Reserve Based Upon Steady-State Computational Fluid Dynamics Analysis: Results From the VIRTU-Fast Study
Source: JACC Basic Transl Sci. 2017 Aug 28;2(4):434–46. doi: 10.1016/j.jacbts.2017.04.003 (PMC5582193; doi:10.1016/j.jacbts.2017.04.003)
Supplement: Supplemental Appendices A–D [file mmc1.pdf]

Appendices

Appendix A: Deriving linear and quadratic coefficients (z_1 and z_2)

The relationship between pressure drop and flow is defined as a second order polynomial:

$$dP = (z_2 \cdot Q^2) + (z_1 \cdot Q) + z_0$$

where dP is pressure gradient,Q is flow and z_2 , z_1, and z_0 are (dimensional) constants.

It is assumed that the pressure drop is zero at zero flow, and so $z_0 = 0$. At two flow rates, Q_1 and Q_2, the pressure drops are computed, and are respectively dP_1 and dP_2.

$$z_1 = \frac{(dP_1 Q_2^2 - dP_2 \cdot Q_1^2)}{(Q_1 Q_2^2 - Q_1^2 Q_2)}$$

$$z_2 = \frac{(dP_1 Q_2 - dP_2 \cdot Q_1)}{(Q_1^2 Q_2 - Q_1 Q_2^2)}$$

Appendix B: Deriving $vFFR_{ps\text{-}trns}$

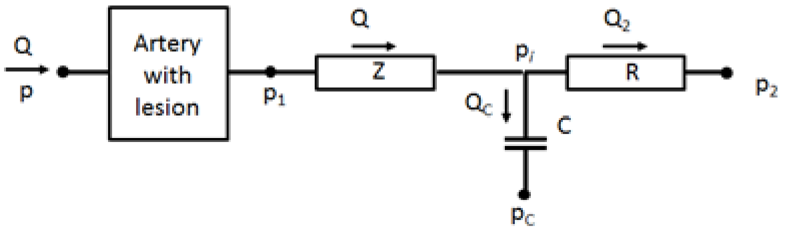

The above diagram illustrates the system that is analysed to compute the Fractional Flow Reserve . The parameters p, p_1, p_i, p_2, p_C, Q, Q_C and Q_2 are all functions of time, p(t) etc. It is assumed that the pressure/flow relationship for the lesion has been characterised as a quadratic equation using the results of two steady-state CFD analyses, with coefficients z_1 and z_2 defined in Appendix A. It is assumed that this formula can be applied to relate the instantaneous pressure drop at any time to

the instantaneous flow rate at that time: the validity of this assumption is tested in the body of this paper. The distal arterial vasculature and microvasculature is assumed to be characterised by a three-element Windkessel as illustrated, and it is assumed that the values of the components of the Windkessel are known for the individual (the tuning of these parameters is in fact a primary challenge in the computation of FFR, but this is not the purpose of this Appendix). The capacitor has a 'back pressure', p_C, applied to represent the pressure generated in the myocardium by the squeezing of the ventricle during systole. It is assumed that this function is known: it might be derived based on assumptions of the relationship between intramyocardial pressure and ventricular pressure, and might be patient-specific (where data to support personalisation is available) or it might be generic. The system thus comprises the nonlinear resistance of the lesion in series with the Windkessel element. The model is further simplified by assuming that the distal pressure $p_2(t)$ representing the venous pressure distal to the Windkessel, is zero. It is assumed that the proximal pressure p(t) is known, by measurement in the individual, by operation of an overall system model or by taking a generic profile from the literature. The proximal resistance of the Windkessel simply operates in series with the characterised lesion and $p_1(t)$ can be recovered as a post processing operation. The system can be solved for the remaining parameters $p_i(t)$, Q(t), $Q_c(t)$ and $Q_2(t)$ as follows.

The inlet flow is related to the pressure drop from the inlet to the internal point within the Windkessel:

$$z_2\mathrm{Q}^2 + z_1\mathrm{Q} + \mathrm{ZQ} = \mathrm{p} - \mathrm{p}_i$$

The flow through the distal resistor of the Windkessel is related to the pressure drop from the internal point to the vein. With p_2=0:

$$\mathrm{RQ}_2 = \mathrm{p}_i$$

The flow onto the capacitor of the Windkessel is:

$$\mathrm{Q}_\mathrm{C} = \mathrm{C}\frac{d(\mathrm{p}_i - \mathrm{p}_\mathrm{C})}{dt}$$

Finally continuity dictates that:

$$\mathrm{Q} - \mathrm{Q}_2 - \mathrm{Q}_\mathrm{C} = 0$$

The above four equations can be combined to produce an equation relating inlet flow to inlet pressure:

Eliminating Q_C: $$\mathrm{Q} - \mathrm{Q}_2 = \mathrm{C}\frac{d(\mathrm{p}_i - \mathrm{p}_\mathrm{C})}{dt}$$

Eliminate Q_2 and p_i: $$\mathrm{Q}_2 = \frac{\mathrm{p}_i}{\mathrm{R}} = \frac{\mathrm{p} - z_2\mathrm{Q}^2 - (z_1 + \mathrm{Z})\mathrm{Q}}{\mathrm{R}} \quad \rightarrow$$

$$\mathrm{Q} - \frac{\mathrm{p} - z_2\mathrm{Q}^2 - (z_1 + \mathrm{Z})\mathrm{Q}}{\mathrm{R}} = \mathrm{C}\frac{d(\mathrm{p} - z_2\mathrm{Q}^2 - (z_1 + \mathrm{Z})\mathrm{Q} - \mathrm{p_C})}{dt}$$

$$\mathrm{C}z_2\frac{d(\mathrm{Q}^2)}{dt} + \mathrm{C}(z_1 + \mathrm{Z})\frac{d\mathrm{Q}}{dt} + \frac{z_2\mathrm{Q}^2}{\mathrm{R}} + \left(1 + \frac{(z_1 + \mathrm{Z})}{\mathrm{R}}\right)\mathrm{Q} = \mathrm{C}\frac{d(\mathrm{p} - \mathrm{p_C})}{dt} + \frac{\mathrm{p}}{\mathrm{R}}$$

And so the final governing equation for the inlet flow, *Q*(t), in terms of the inlet pressure, the intramyocardial pressure and the system parameters (lesion and Windkessel) is:

$$\frac{2z_2}{(z_1 + \mathrm{Z})}\mathrm{Q}\frac{d\mathrm{Q}}{dt} + \frac{d\mathrm{Q}}{dt} + \frac{z_2\mathrm{Q}^2}{(z_1 + \mathrm{Z})\mathrm{RC}} + \left(\frac{z_1 + \mathrm{Z} + \mathrm{R}}{(z_1 + \mathrm{Z})\mathrm{RC}}\right)\mathrm{Q} = \frac{1}{z_1 + \mathrm{Z}}\frac{d(\mathrm{p} - \mathrm{p_C})}{dt} + \frac{\mathrm{p}}{(z_1 + \mathrm{Z})\mathrm{RC}}$$

Numerical Solution

Using a simple explicit solution method (first order forward Euler) to solve for *Q* given p, with Q_j indicating the solution for *Q* at time *j*:

$$\left(\frac{2z_2}{(z_1 + \mathrm{Z})}\mathrm{Q}_j + 1\right)\frac{(\mathrm{Q}_{j+1} - \mathrm{Q}_j)}{\mathrm{dt}} + \frac{z_2{\mathrm{Q}_j}^2}{(z_1 + \mathrm{Z})\mathrm{RC}} + \left(\frac{z_1 + \mathrm{Z} + \mathrm{R}}{(z_1 + \mathrm{Z})\mathrm{RC}}\right)\mathrm{Q}_j = \frac{1}{(z_1 + \mathrm{Z})}(\mathrm{dp})_j - \frac{1}{(z_1 + \mathrm{Z})}(\mathrm{dp_C})_j + \frac{\mathrm{p}_j}{(z_1 + \mathrm{Z})\mathrm{RC}}$$

where: $(\mathrm{dp})_j = \left(\frac{d\mathrm{p}}{dt}\right)_j$; $(\mathrm{dp_C})_j = \left(\frac{d\mathrm{p_C}}{dt}\right)_j$

$$\mathrm{Q}_{j+1} = \mathrm{Q}_j + \frac{\mathrm{dt}}{\frac{2z_2}{(z_1 + \mathrm{Z})}\mathrm{Q}_j + 1}\left(-\frac{z_2{\mathrm{Q}_j}^2}{(z_1 + \mathrm{Z})\mathrm{RC}} - \left(\frac{z_1 + \mathrm{Z} + \mathrm{R}}{(z_1 + \mathrm{Z})\mathrm{RC}}\right)\mathrm{Q}_j + \frac{((\mathrm{dp})_j - (\mathrm{dp_C})_j)}{(z_1 + \mathrm{Z})} + \frac{\mathrm{p}_j}{(z_1 + \mathrm{Z})\mathrm{RC}}\right)$$

Thus the inlet flow at every time step can be computed, and then the pressures can be recovered from the appropriate resistance equation. The inlet pressure p(t) and the pressure distal to the lesion, p_1(t) can be averaged over the cardiac cycle to compute the FFR.

Appendix C: Deriving $\mathrm{vFFR_{steady}}$

The equations can be simplified further if it is assumed that the transient effects over the cardiac cycle can be neglected for the computation of FFR (which is a time-averaged measure). Then it is assumed that all parameters are independent of time and:

$$z_2\mathrm{Q}^2 + z_1\mathrm{Q} + \mathrm{ZQ} \ = \mathrm{p} - \mathrm{p}_i$$

$$\mathrm{RQ} \ = \mathrm{p}_i$$

$$z_2\mathrm{Q}^2 + z_1\mathrm{Q} + \mathrm{ZQ} \ = \mathrm{p} - \mathrm{RQ}$$

$$z_2\mathrm{Q}^2 + (z_1 + \mathrm{Z} + \mathrm{R})\mathrm{Q} - \mathrm{p} \ = 0$$

$$\mathrm{Q} \ = \frac{-(z_1 + \mathrm{Z} + \mathrm{R}) + \sqrt{(z_1 + \mathrm{Z} + \mathrm{R})^2 + 4z_2\mathrm{p}}}{2z_2}$$

Appendix D: Global sensitivity Analysis

The suggested variance decomposition in the Sobol decomposition can be written as:

$$Var(y) = \sum_i V_i + \sum_i \sum_{j>i} V_{ij} + \cdots + V_{12\ldots k} \qquad (1.1)$$

With V_i the variance due to input parameter X_i alone, V_{ij}(interaction effect terms) the variance due to interaction effects between input parameters X_i and X_j and so on. Normalising eq. 1.1 by the model variance $Var(y)$ result in an expression containing the sensitivity indices (right hand side eq 1.2):

$$\frac{Var(y)}{Var(y)} = 1 = \sum_i S_i + \sum_i \sum_{j>i} S_{ij} + \cdots + S_{12\ldots k} \qquad (1.2)$$

The aim of the sensitivity analysis is to provide a main sensitivity index, S_i, and a total sensitivity index S_i^T, for each input parameter x_i, which are defined in eq. 1.3-1.4 below.

$$S_i = \frac{Var(E(y|x_i))}{Var(y)} \qquad (1.3)$$

$$S_i^T = \frac{E(Var(y|x_{-i}))}{Var(y)} = 1 - \frac{Var(E(y|x_{-i}))}{Var(y)} \quad (1.4)$$

If the true value of a parameter was known, the variance of your model would decrease by a proportion S_i which is apparent from equation 1.2. It can therefor be used to quantify the importance of the direct effects of a model input parameter.

The total sensitivity index of an input parameter is the sum of the main sensitivity index and all sensitivity indices due to interaction effect terms dependent on this input parameter. As an example the terms are written out for a model containing 3 input parameters and 1 output in equation 1.5. Note that the sum of all total sensitivity indices will always exceed 1.

$$S_i^T = S_1 + S_{12} + S_{13} + S_{123} \quad (1.5)$$

A low value of the total sensitivity index implies that variation of a particular input parameter has no effect on the variation of the output. Therefore this index can be used to determine which parameters can be fixed to population averages.
